# RETRACTION: The Cell Surface GRP78 Facilitates the Invasion of Hepatocellular Carcinoma Cells

**DOI:** 10.1155/bmri/9783436

**Published:** 2025-04-29

**Authors:** BioMed Research International

**RETRACTION**: X. Zhang, H. Li, S. Zhao, L. Zhao, H. Song, G. Wang, Q. Guo, Z. Luan, R. Su, “The Cell Surface GRP78 Facilitates the Invasion of Hepatocellular Carcinoma Cells,” *BioMed Research International*, 2013, https://doi.org/10.1155/2013/917296

The above article, published online on 08 December 2013 in Wiley Online Library (http://wileyonlinelibrary.com), has been retracted by agreement between the authors, the Handling Editor, Dr. Paul J. Higgins; and John Wiley & Sons Ltd. The retraction has been agreed following an investigation into figure concerns initially raised on PubPeer [1], and was requested by the authors. Specifically:

• In Figure 1a, the GAPDH PLC panel appears to be identical to the Figure 3a GAPDH deltaKDEL panel.
• In Figure 2a, the IgG panel appears to contain unexpectedly repeated elements when compared to the Figure 3e deltaKDEL panel.
• In Figure 2a, the UT panel appears to contain unexpectedly repeated elements when compared to the Figure 2b UT panel.
• In Figure 2a, the N20 panel appears to contain unexpectedly repeated elements when compared to the Figure 2b N20 panel.
• In Figure 2b, the UT panel appears to be identical to the Figure 3e mock panel.

The authors were contacted and explained these errors were introduced during the figure preparation, however, the response did not satisfy our concerns and it is therefore retracted.

The authors agree to the retraction and the notice.
